# Prognostic factors of septic shock

**DOI:** 10.1186/cc10658

**Published:** 2012-03-20

**Authors:** JP Quenot, A Pavon, C Binquet, F Kara, O Martinet, F Ganster, JC Navellou, V Castelain, D Barraud, J Cousson, JF Poussel, P Perez, K Kuteifan

**Affiliations:** 1 University Hospital Bocage, Dijon, France; 2 CIC EC, Dijon, France; 3 Centre Hospitalier, Haguenau, France; 4 Nouvel Hopital Civil, Strasbourg, France; 5 University Hospital, Besancon, France; 6 Hopital Hautepierre, Strasbourg, France; 7 Hopital Central, Nancy, France; 8 Regional Hospitalier, Metz-Thionville, France; 9 Hopital Brabois, Nancy, France

## Introduction

The incidence of septic shock in intensive care (ICU) in France is around 8 to 10%, with in-hospital mortality ranging from 55 to 60% [1]. The identification of prognostic factors is essential to guarantee optimal management.

## Methods

A prospective, multicentre, observational study was performed between November 2009 and March 2011 in 14 ICUs from 10 university and community (nonacademic) hospitals in the northeast of France. This study was supported by the Collège Interrégional des Réanimateurs du Nord-Est. Patients were included if they were aged >18 years and had septic shock plus at least one criterion of hypoperfusion. Data control and statistical analysis was performed by the CIC-EC of Dijon University Hospital (INSERM Unit CIE1). Univariate and multivariate logistic regression analysis was used to identify predictors of mortality at 28 days.

## Results

In total, out of 7,833 patients admitted to intensive care during the study period, 1,147 (14.6%) had septic shock. Factors significantly associated with mortality at 28 days by logistic regression were: age >70 (OR 1.98, 95% CI 1.5 to 2.6, *P *< 0.01); transfer (OR 1.42, 95% CI 1.04 to 1.95, *P *= 0.02); immunodepression (OR 1.91, 95% CI 1.41 to 2.57, *P *< 0.01); Knaus score C-D (OR 2.16, 95% CI 1.64 to 2.84, *P *< 0.01); SOFA score (OR for an increase of 1 point 1.32, 95% CI 1.26 to 1.38, *P *< 0.01); and infection acquired in the ICU (OR 1.86, 95% CI 1.03 to 3.37, *P *= 0.03). Protective factors were surgical admission (OR 0.61, 95% CI 0.41 to 0.89, *P *= 0.01) and urinary tract infection (OR 0.55, 95% CI 0.37 to 0.82, *P *< 0.01).

## Conclusion

Our findings are coherent with the literature. Multivariate analysis found nonmodifiable risk factors such as age, but also modifiable risk factors that warrant further investigation, such as infections acquired in-hospital or in the ICU. Future clinical studies in septic shock should take these findings into account when selecting patients.
